# Lymphocyte subset phenotyping for the prediction of progression to inflammatory arthritis in anti-citrullinated-peptide antibody-positive at-risk individuals

**DOI:** 10.1093/rheumatology/kead466

**Published:** 2023-09-07

**Authors:** Innocent Anioke, Laurence Duquenne, Rekha Parmar, Kulveer Mankia, Farag Shuweihdi, Paul Emery, Frederique Ponchel

**Affiliations:** Leeds Institute of Rheumatic and Musculoskeletal Medicine, University of Leeds, Leeds, UK; Department of Medical Laboratory Sciences, Enugu Campus, University of Nigeria, Enugu State, Nigeria; Leeds Institute of Rheumatic and Musculoskeletal Medicine, University of Leeds, Leeds, UK; NIHR Leeds Biomedical Research Centre, Leeds Teaching Hospitals NHS Trust, Leeds, UK; Leeds Institute of Rheumatic and Musculoskeletal Medicine, University of Leeds, Leeds, UK; Leeds Institute of Rheumatic and Musculoskeletal Medicine, University of Leeds, Leeds, UK; NIHR Leeds Biomedical Research Centre, Leeds Teaching Hospitals NHS Trust, Leeds, UK; Leeds Institute of Health Sciences, University of Leeds, School of Medicine, Leeds, UK; Leeds Institute of Rheumatic and Musculoskeletal Medicine, University of Leeds, Leeds, UK; NIHR Leeds Biomedical Research Centre, Leeds Teaching Hospitals NHS Trust, Leeds, UK; Leeds Institute of Rheumatic and Musculoskeletal Medicine, University of Leeds, Leeds, UK

**Keywords:** lymphocytes, RA, rheumatic diseases, immunological techniques, biomarkers, laboratory diagnosis, T cells

## Abstract

**Objectives:**

Inflammatory arthritis (IA) is considered the last stage of a disease continuum, where features of systemic autoimmunity can appear years before clinical synovitis. Time to progression to IA varies considerably between at-risk individuals, therefore the identification of biomarkers predictive of progression is of major importance. We previously reported on the value of three CD4+T cell subsets as biomarkers of progression. Here, we aim to establish the value of 18 lymphocyte subsets (LS) for predicting progression to IA.

**Methods:**

Participants were recruited based on a new musculoskeletal complaint and being positive for anti-citrullinated-peptide antibody. Progression (over 10 years) was defined as the development of clinical synovitis. LS analysis was performed for lymphocyte lineages, naive/memory subsets, inflammation-related cells (IRC) and regulatory cells (Treg/B-reg). Modelling used logistic/Cox regressions.

**Results:**

Of 210 patients included, 93 (44%) progressed to IA, 41/93 (44%) within 12 months (rapid progressors). A total of 5/18 LS were associated with progression [Treg/CD4-naïve/IRC (adjusted *P* < 0.0001), CD8 (*P* = 0.021), B-reg (*P* = 0.015)] and three trends (NK-cells/memory-B-cells/plasmablasts). Unsupervised hierarchical clustering using these eight subsets segregated three clusters of patients, one cluster being enriched [63/109(58%)] and one poor [10/45(22%)] in progressors. Combining all clinical and LS variables, forward logistic regression predicted progression with accuracy = 85.7% and AUC = 0.911, selecting smoking/rheumatoid-factor/HLA-shared-epitope/tender-joint-count-78 and Treg/CD4-naive/CD8/NK-cells/B-reg/plasmablasts. To predict rapid progression, a Cox regression was performed resulting in a model combining smoking/rheumatoid factor and IRC/CD4-naive/Treg/NK-cells/CD8+T cells (AUC = 0.794).

**Conclusion:**

Overall, progression was predicted by specific LS, suggesting potential triggers for events leading to the development of IA, while rapid progression was associated with a different set of subsets.

Rheumatology key messages

- Novel predictive value is associated with B-reg, NK-cells-CD56^bright^ and CD8+T-cells in addition to Treg/naive-CD4+T-cells.
- Different lymphocyte subset profiles are associated with clusters of patients with different IA outcomes.
- Overall and short-term progression are predicted by different cell subsets and clinical variables.

## Introduction

RA is a chronic autoimmune, inflammatory joint disease. A pre-clinical phase of RA has been identified, also known as the at-risk phase of the inflammatory arthritis continuum (IAC) [1, 2]. The at-risk phase can last up to 15 years, during which genetic and environmental factors contribute to the progression including a break in tolerance and the development of systemic autoimmunity manifested by the presence of autoantibodies (particularly, anti-citrullinated peptide antibodies-ACPA). The development of pain and other musculoskeletal symptoms in the absence of synovitis precedes a final stage when synovitis develops. Treatment is conventionally initiated upon the detection of clinical synovitis.

Given the success of treatment for early RA [3], predicting an individual’s progression to IA/RA may enable preventive interventions. However, the rate of progression to IA is ∼30–40% depending on the criteria used to identify at-risk individuals [4, 5]. ACPA and/or rheumatoid factor (RF) are widely used to identify such individuals, and various prediction models were established by combining demographic/genetic/clinical/imaging data [6, 7].

The involvement of immune cells in RA pathogenesis has been extensively described (T/B/NK-cells, monocytes [8–13]). We established the predictive value of the frequencies of circulating naive CD4+T cells, regulatory T cells (Tregs), and a subset of naive cells (CD62L-) able to enter IL6-expressing tissues (called inflammation-related cells IRC) for RA diagnosis, for the induction of remission at first treatment, flares in patients in clinical remission and ability to safely discontinue anti-TNF [14–16]. In ACPA+ at-risk individuals, these three CD4+T-cell subsets showed good predictive value as biomarkers for progression to IA, both individually and when combined with clinical variables [15, 17].

The specific cellular and molecular events that influence progression to the next stage of the IAC [18] remain unclear. Here, we test the hypothesis that the dysregulation of other lymphocyte subsets (LS), in addition to the three CD4+T-cell subsets previously reported [15, 17], may provide mechanistic clues as to the cellular events underpinning the progression to IA. Furthermore, we proposed to investigate whether an extended LS analysis (performed by hospital services on fresh blood over a few hours), can provide an improvement of the performances of current prediction models [15, 17] that are using only three CD4+T-cell subsets in combination with demographic and clinical data.

## Patients and methods

### Study cohort

Patients have been recruited in the Coordinated Programme to Prevent Arthritis register since 2008. Ethical approval was obtained (REC approval: 06/Q1205/169) and all participants provided informed written consent.

Recruitment and follow-up have been described in a previous publication [6]. To sum up, recruitment criteria were a new non-specific musculoskeletal complaint and ACPA (detected by a routine hospital test) or RF positivity or as a relative to patients with RA, but in the absence of clinical synovitis. Participants were followed until IA occurred. Progression is defined by the development of inflammatory arthritis (clinical synovitis) i.e. ≥1 swollen joint and evaluated by a senior rheumatologist. Patients with non-progression were included only if they had >12 months of follow-up (details in supplementary material, available at *Rheumatology* online). Data collected included demographic (age, gender), lifestyle (smoking, alcohol), genetic (shared epitope, HLA-SE), physical assessment (78 joint tenderness, early morning stiffness), serology (RF) and inflammatory markers (erythrocyte sedimentation rate, C-reactive protein).

Patients were selected from the register (which recruited over 500 participants) if they had flow cytometry performed (see below) and if they were ACPA-positivity by a second generation CCP-2 test (Immuno-CAP, Phadia, Sweden, positivity cut-off at 10 OD), performed in our research lab as false positivity with the hospital test was recently described [19], and irrespective of positivity for RF.

All patients included in this study (*n* = 210), had data for the CD4+T cells panel (naive, IRC) the lymphocyte count panel (LS), and the Treg panels’ missingness is described. Some were excluded due to technical issues (poor quality of blood due to transport delays), while for the Treg panel, a shortage of one antibody (FoxP3) led to the panel not being done. For the CD8 panel, delays in transport were associated with difficulties in gating the CD8-IRC subset. The B-cell panel was introduced late (2015), hence limiting us to 210 patients among all participants in the register. Altogether, 150/210 patients had a full dataset and we imputed data for individual subsets in 41 patients with >15/18 LS present. We inputted data for the B-panel in 19 patients who had complete data for all other LS.

LS were quantified by flow cytometry using five panels for lineage: CD4+/CD8+ T-cell subsets (naive, memory, IRCs); Treg; B-cell subsets (naive, memory, plasmablasts, B-reg). Detailed methodology is described in supplementary material (available at *Rheumatology* online) and previously detailed [15]. All blood samples were processed fresh within a few hours of the collection after transport from the clinics to the central NHS immunology laboratory in Leeds. The gating strategies are illustrated in Supplementary Fig. S1, available at *Rheumatology* online. LS frequencies were reported as the percentage of the parental population. Age relationships for specific cell subsets were previously described [20] and normalisation was applied [15].

### Statistical analysis

A Spearman correlation-based clustering algorithm (Cluster-3, Stanford University 1998–99) was applied after log transformation to assess collinearity between LS frequencies. A heat-map was generated using TreeView.

Exploration of data was performed for univariate analysis using Mann–Whitney U and χ^2^ tests, between progressors and non-progressors groups. Correction for multiple testing was applied in univariate analysis using Bonferroni correction [21, 22]. Variables were assessed individually for predictive value using the unadjusted odd ratio (OR, 95%Cl) and area under the ROC curve (AUC, 95%Cl). Logistic and Cox regression modelling was used to predict progression using a stepwise forward method [23], selecting the best combination of variables for significant improvement of the fit. Missing data imputation was performed using the tool for multiple imputation process in SPSS setting up the boundaries of data to be inputted and then the ‘Imputing Missing Data Values’ function over five cycles. The estimates obtained from each dataset (five imputation datasets) were aggregated to produce an overall imputation estimate using the same SPSS package. The pooled dataset after imputation was compared with the non-imputed dataset to verify that ORs were not affected by the imputation process. Pooled analysis was performed, and ORs were not affected by the imputation process. Sensitivity, specificity and positive/negative predictive values (PPV/NPV) were calculated from the classification matrix of accurately predicted cases obtained from logistic regression. A total of 14% of cases had one or another missing data, while we confirmed that data were missing at random using Little’s MCAR test (*P* < 0.0001). WALD tests from the regression analyses performed were used to assess the contribution of each of our predictor variables.

A bootstrapping technique was used (500 permutations) to calculate a discrimination index correcting for optimism between the predicted and actual outcome for the best regression models.

We used graphical diagnostics based on the scaled Schoenfeld residuals to examine the proportional assumption for the Cox regression. This allowed us to assess whether the hazard functions remained proportional over time.

Data were then analysed using the SPSS V27, R (V4.1.3); *P*-values of <0.05 were considered significant.

## Results

### Cohort outcome

ACPA+ patients (*n* = 210) with a minimum of 12 months (up to 10 years) of follow-up were selected from the overall cohort. Clinical data were retrieved and progression to clinical synovitis was observed in 93/210 (44%) of participants, occurring under 12 months in 41 patients (rapid progression), within 13–24 months for 18 patients, with the last 33 progressing later than 2 years post inclusion and one patient after 10 years (Supplementary Fig. S2, available at *Rheumatology* online). A total of 75% of progressors met the EULAR 2010 Classification criteria for RA at the time of progression with an average of >3.5 swollen joints (range 1–15) and were directed to our early arthritis clinic for further care.

Demographic/clinical data are described in Table 1. Association between progression and data at inclusion suggested two highly significant parameters (HLA-SE/RF, MWU *P* < 0.0001 after correction) and another three potential associations (smoking/EMS/TJC78, MWU *P* < 0.05), consistent with published reports in this cohort [6]. AUCs were calculated suggesting high values for RF/HLA-SE (AUC > 0.650, *P* < 0.0001) and for smoking/TJC78/EMS (AUC > 0.600, *P* < 0.010). The individual contribution to the prediction was however relatively small for all parameters with 19% for RF (Wald test) and >8% for the other three variables. Of note, no difference in ACPA levels were detected between progressors (mean 335 OD) or non-progressors (341 OD) as well as between rapid (355 OD) and delayed (344 OD) progressors.

**Table 1.** Association of demographic and clinical data with progression (*n* = 210)

	Progressors *n* = 93 (44.3%)	Non-progressors *n* = 117 (55.7%)	Adjusted *P*-value^b^	AUROC (95%CI) *P*-value	Unadjusted OR (95%CI) *P*-value	Wald test
Age (years)^a^	53.0 (43,63)	51.0 (42,61.)	0.292	0.542 (0.464–0.620) 0.292	1.013 (0.993–1.033) 0.217	1.5
Gender (Female)	60 (64.5%)	87 (74.4%)	0.132	0.549 (0.470–0.628) 0.221	1.595 (0.881– 0.888) 0.123	2.4
Alcohol (unit)	4.40 (0.0,10.15)	4.50 (0.0,9.80)	0.327	0.461 (0.383–0.540) 0.335	0.994 (0.974–1.014) 0.564	0.3
Smoking Never Ever	23 (24.7%) 70 (75.3%)	53 (45.3%) 64 (54.7%)	0.002	0.603 (0.526–0.679) 0.011	2.520 (1.390–4.571) 0.002	9.3
HLA-SE [positive]	68 (73.1%)	56 (47.9%)	<0.0001	0.626 (0.551–0.702) 0.002	2.963 (1.651– 5.316) <0.0001	13.3
RF [Positive]	57 (61.3%)	35 (29.9%)	<0.0001	0.657 (0.582–0.732) <0.0001	3.710 (2.087–6.593) <0.0001	19.9
ESR (mm/h)^a^	14 (6.5,20.00)	12.0 (7.0,20.5)	0.343	0.538 (0.459–0.617) 0.344	1.019 (0.993–1.045) 0.162	1.9
CRP (mg/L)^a^	3.180 (0.99,6.795)	3.00 (0.57,5.76)	0.159	0.557 (0.479–0.635) 0.159	1.044 (0.995–1.096) 0.082	3.0
EMS (min)^a^	22 (0.,60.)	5 (0,30.)	0.007	0.604 (0.527–0.681) 0.009	1.007 (1.000–1.014) 0.044	4.0
TJC78^a^	1 (0,3)	1 (0,2.)	0.009	0.601 (0.524–0.678) 0.012	1.113 (0.992–1.249) 0.068	3.3

Categorical data are presented as *n* (% of participant).

a Numerical data are presented as median (Interquartile range values); MWU Mann-Whitney U and χ^2^ tests for continues and categorical variables, respectively, were used.

b Tests adjusted for 10 comparisons (adjustment of the *P-value* was performed by applying Bonferroni correction method for multiple comparison test).

AUROC: area under the roc curve; EMS: early morning stiffness; HLA (SE): human leucocyte antigen (shared epitope); TJC: tender joint count.

### Flow cytometry analysis and univariate association with progression

**Figure 1.**
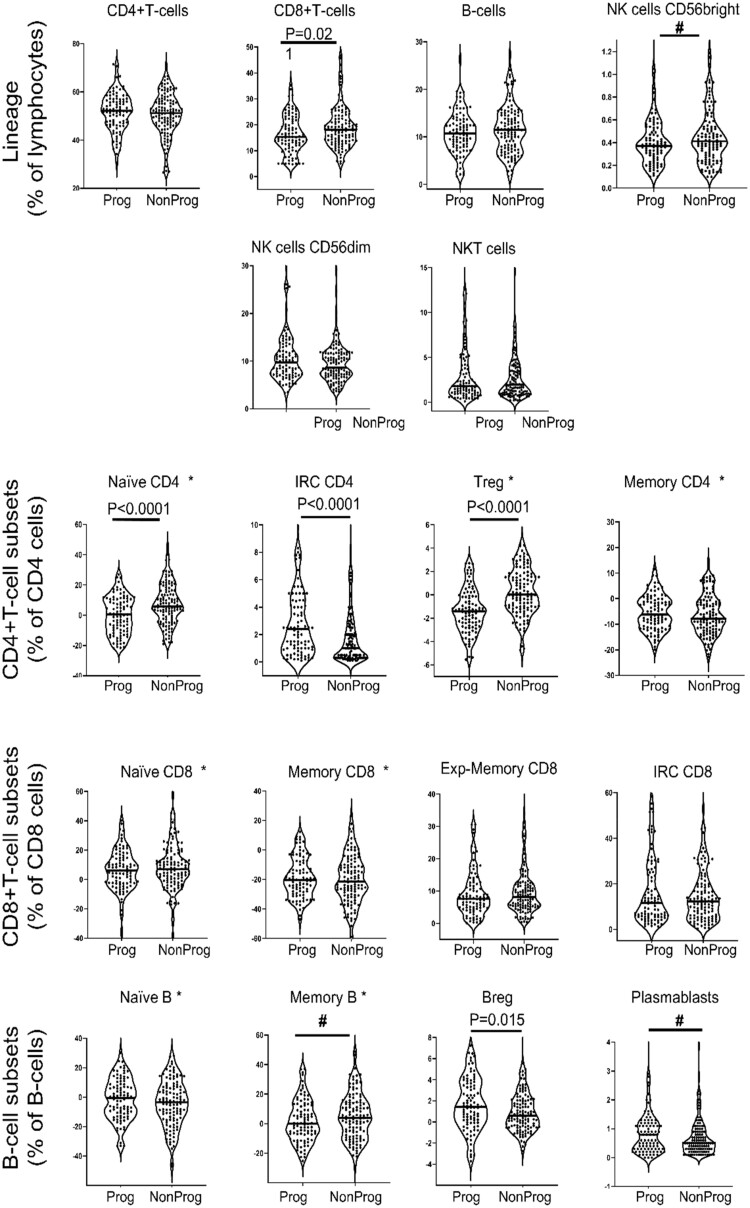
Frequency of lineage and lymphocytes subsets in at-risk progressors *vs* non-progressors. LS were analysed by flow cytometry and data displayed as violin plots (each dot representing a patient) for Progressor (Prog, *n* = 93) and non-Progressors (Non-Prog, *n* = 117). Star (*) indicates LS that were normalised as previously described [15]. *P-*value corrected for multiple testing (MWU test) are indicated when significant and # designate trends

The overall results of LS phenotyping (Fig. 1, Table 2) showed significant association with progression for five subsets: lower naive CD4+T cells, Treg (*P* < 0.0001) and CD8+T cells (*P* = 0.021), and higher CD4-IRC (*P* < 0.0001) and B-reg (*P* = 0.015). Three more subsets, higher NK-cells CD56^bright^, higher memory B cells and lower plasmablasts showed non-significant difference (after correction) that may nonetheless suggest other biological events leading to progression.

**Table 2.** Association of 18 LS with progression (*n* = 210)

	Missing data *n* (%)	Progressors *n* = 93 (44.3%)	Non-progressors *n* = 117 (55.7%)	Adjusted *P*-value^a^	AUROC (95%CI) *P*-value	Unadjusted OR (95%CI) *P-*value	Wald test
CD4 T cells	none	52.11 (46.41 56.96)	51.18 (45.98 56.35)	0.479	0.528 (0.450–0.607) 0.479	1.014 (0.982–1.048) 0.394	0.7
CD8 T cells	19 (9%)	15.34 (11.57 21.97)	17.98 (13.68 23.46)	0.021	0.407 (0.330–0.485) 0.021	0.952 (0.917–0.988) 0.009	6.8
B cells	none	10.73 (7.9713.55)	11.44 (7.41 14.66)	0.503	0.473 (0.395–0.551) 0.503	0.972 (0.917–1.030) 0.337	0.9
NK cells CD56^b^^right^	none	0.37 (0.27 0.53)	0.41 (0.27 0.57)	0.154	0.447 (0.369–0.524) 0.164	0.485 (0.178–1.317) 0.156	2.0
NK cells CD56^dim^	none	9.69 (7.10 12.53)	8.61 (6.85 11.67)	0.207	0.551 (0.472–0.629) 0.205	1.052 (0.987–1.120) 0.117	2.5
NKT cells	none	1.78 (1.01 4.16)	1.93 (0.96 3.87)	0.878	0.506 (0.427–0.586) 0.878	1.021 (0.943–1.106) 0.610	0.2
Naïve CD4 cells^b^	13 (6.5%)	0.42 (−10.10 10.58)	5.74 (−2.59 16.38)	<0.0001	0.355 (0.280–0.429) <0.0001	0.956 (0.934–0.978) <0.0001	14.8
Memory CD4 cells^b^	23 (11%)	−6.23 (−11.34–0.38)	−7.81 (−11.93–1.17)	0.327	0.539 (0.461–0.618) 0.327	1.016 (0.979–1.055) 0.407	0.7
IRC CD4 cells	13 (6.5%)	2.40 (1.00 4.50)	1.00 (0.30 2.50)	<0.0001	0.658 (0.584–0.732) <0.0001	1.245 (1.098–1.411) <0.0001	11.7
Treg CD4 cells^b^	20 (9.3)	−1.41 (−2.71–0.13)	0.01 (−1.10 1.64)	<0.0001	0.273 (0.205–0.342) <0.0001	0.651 (0.554–0.765) <0.0001	27.0
Naïve CD8 cells^b^	35 (16.5%)	6.16 (−2.60 14.78)	6.91 (−0.82 14.78)	0.448	0.469 (0.391–0.548) 0.448	0.992 (0.973–1.011) 0.387	0.7
Memory CD8 cells^b^	36 (17%)	−20.22 (−28.92–7.65)	−21.32 (−30.83–9.72)	0.533	0.525 (0.477–0.603) 0.533	1.006 (0.988–1.024) 0.516	0.4
Exp-memory like CD8 cells	39 (19%)	7.60 (4.46 12.40)	8.20 (5.32 12.40)	0.296	0.458 (0.379–0.537) 0.296	0.980 (0.945–1.017) 0.281	1.2
IRC CD8 cells	37 (18%)	11.50 (5.55 25.09)	12.20 (5.60 19.90)	0.959	0.498 (0.418–0.578) 0.959	1.006 (0.986–1.027) 0.536	0.4
Naive B cells^b^	19 (12.6%)	−0.50 (−11.19 8.83)	−3.39 (−15.106.94)	0.190	0.554 (0.476–0.632) 0.189	1.014 (0.995–1.033) 0.179	2.2
Memory B cells^b^		0.10 (−7.35 12.42)	4.05 (−7.89 14.42)	0.135	0.440 (0.362–0.518) 0.138	0.986 (0.967–1.004) 0.125	2.4
Regulatory B cells		1.42 (−0.05 3.56)	0.60 (−0.47 2.05)	0.015	0.598 (0.520–0.677) 0.015	1.164 (1.021–1.325) 0.021	5.3
Plasmablasts		0.80 (0.30 1.20)	0.50 (0.30 0.95)	0.105	0.565 (0.486–0.644) 0.106	1.119 (0.873–1.432) 0.175	0.8

Data are presented as median (interquartile range values).

a MWU Mann–Whitney *U* test adjusted *P*-value for 18 comparison (adjustment of the *P*-value was performed by applying Bonferroni correction method for multiple comparison test).

b Normalised subsets.

AUROC: area under the roc curve; Exp-: expanded; IRC: inflammatory-related cells; NK: natural killer; NKT: natural killer-T; Treg: regulatory T cells.

AUCs were calculated and the same eight LS had significant/possible predictive values for progression. Of note, Treg, naive CD4+T cells and IRC showed the highest contribution to the prediction (Table 2, Wald score 27%, 15% and 12%, respectively) while each, >6% for CD8, CD56, Breg and plasmablasts. These subsets had high specificity for progression (all >80%) but relatively low sensitivities (32–40%), except for CD4+Treg (60%) (Supplementary Table S1, available at *Rheumatology* online).

There was only one significant correlation between two subsets (naive B cells/memory B cells, rho = −0.885, *P* < 0.0001).

### Clusters of patients

To define groups of patients with similar LS profiles, we used hierarchical clustering, while not specifying the outcome (unsupervised). A pilot analysis using heat-map of frequencies (Supplementary Fig. S3, available at *Rheumatology* online), showed three distinct distributions of the LS. Each group contained one of the highly predictive subsets, suggesting that these were dysregulated independently of each other. The analysis also segregated patients into three clusters with different proportions of progressors (*P* < 0.0001) suggesting that different LS profiles can discriminate patients with different outcomes.

**Figure 2.**
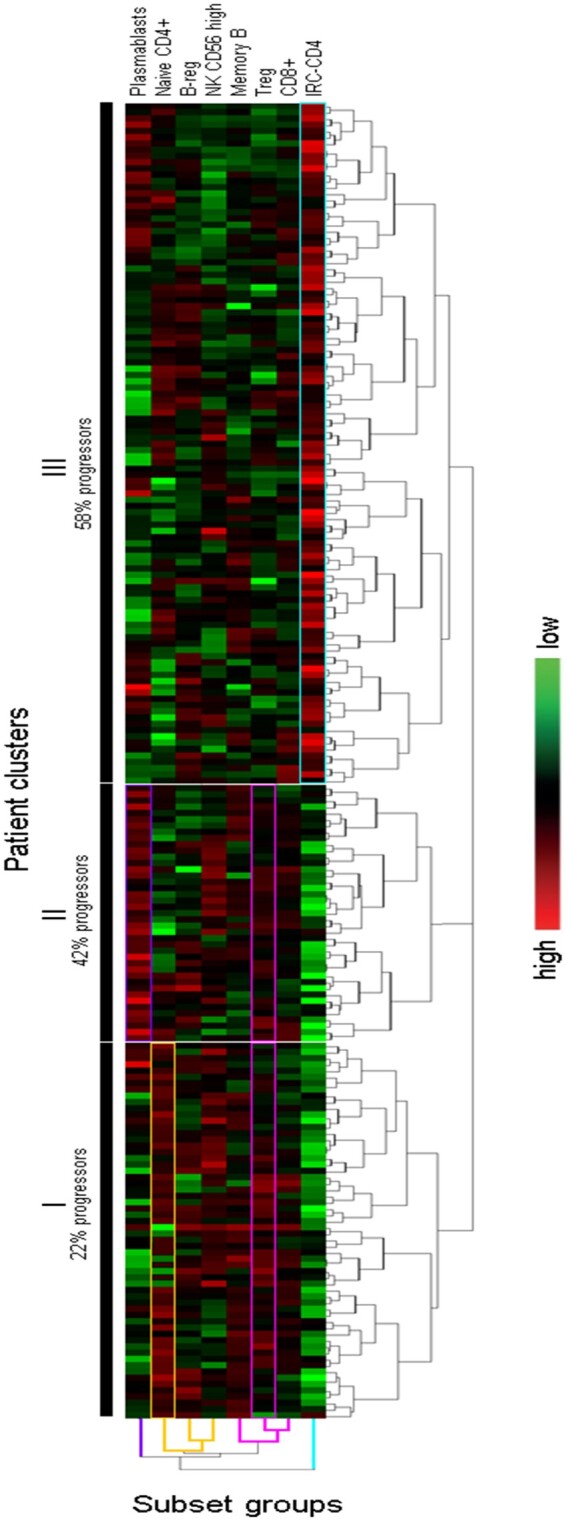
Unsupervised hierarchical clustering of the 8 subsets associated with progression to IA (*n* = 210). An unsupervised hierarchical clustering algorithm was applied to log transformed frequencies for 8 LS and results are displayed as a heat-map of data. This clustering algorithm builds relationships between LS frequencies based on spearman rank correlations, and segregated patients into 3 clusters (I, II and III), annotated with the % of progressors. The first group of LS (plasmablasts only) shows particularly high frequencies in patient cluster-II . The 2nd group with 3 subsets (naïve CD4+T cells, B-reg, and NK CD56high) defined Cluster-II. The third group with 3 subsets (Memory B-cells, Treg and CD8+T cells) allows to define both Cluster I and II. The last group (IRC-CD4+T only) shows exclusively high frequencies in cluster-III with lower frequencies in Cluster-I and II. The proportion of progressors to IA in the 3 clusters was significantly different (*P* < 0.0001). The bar with shades of colours (right hand-side) indicated the frequency observed for each LS from highest to the lowest

We repeated this approach limiting data to the eight subsets identified above. This analysis distributed LS in four groups and patients in three clusters (Fig. 2). The first subset LS group (purple) included only plasmablasts, the second (orange) included naive CD4+T, B-reg and NK-CD56^bright^ cells; the third (pink) combined memory B cells, Treg and CD8+T cells; the last group (blue) with IRC-CD4 alone. Cluster-I was defined by high Treg and high naive CD4 but very low IRC-CD4 and was mainly composed of non-progressors 47/60 (78%). Cluster-II was mainly driven by high plasmablasts while Tregs were also high and CD4+IRC low. This profile was associated with a mixed outcome of 17/41 (42%) progressors and 24/41 (58%) non-progressors. In the largest cluster-III, IRC-CD4 were particularly high and all other LS showed mixed patterns defining subgroups of patients. The proportion of progressors in cluster III was 58% (63/109), which was significantly different from the other two clusters (*P* < 0.0001).

There was no difference in any demographic or clinical data between these three clusters of patients. We, however, observed higher levels of ACPA (*P* = 0.012) in cluster III (CCP2-test: mean 220 OD) compared with cluster I (158 OD,) but not with cluster II (243 OD, *P* = 0.177). No association was seen between plasmablasts and ACPA (or RF) levels.

### Multivariate modelling for the prediction of progression to IA

We previously reported predictive value for three CD4+T cell subsets (naive/IRC/Treg) [15]. Modelling using an enter approach in these 210 patients (as in previous work, Supplementary Table S2, available at *Rheumatology* online) [15], confirmed previous data with an accuracy = 78.6% with an AUC = 0.880, although IRC did not independently contribute to this model (*P* = 0.144).

We then used logistic regressions with a forward approach (Table 3) to determine the predictive value of the demographic/clinical data alone (model-1), of the LS alone (model-2) and of the combination of both datasets (model-3).

**Table 3.** Modelling for the predicting of overall and rapid progression

	Logistic regression OR (95% CI) *P*-value (Wald test) *n* = 210				COX regression HR (95% CI) *P*-value (Wald test) *n* = 158			
	Unadjusted	Model 1	Model 2	Model 3	Unadjusted	Model 4	Model 5	Model 6
Smokers (ever)	2.520 (1.390–4.571) 0.002 (9.3)	2.282 (1.1.187–4.388) 0.013 (6.1)	Not included in the model	3.158 (1.264–7.891) 0.014 (6.1)	2.005 (1.250–3.216) 0.004 (8.3)	2.662 (1.249–5.673) 0.011 (6.5)	Not included in the model	3.688 (1.685–8.074) 0.001 (10.7)
HLA-SE positive	2.963 (1.651– 5.316) <0.0001 (13.3)	2.527 (1.335–4.782) 0.004 (8.1)		2.871 (1.212–6.800) 0.017 (5.7)	2.039 (1.288–3.228) 0.002 (9.2)	Not retained		Not retained
RF positive	3.710 (2.087–6.593) <0.0001 (19.9)	3.600 (1.947–6.656) <0.0001 (16.7)		3.890 (1.681–9.004) 0.002 (10.0)	2.659 (1.743–4.056) <0.0001 (20.6)	4.767 (2.365–9.610) <0.0001 (18.9)		4.784 (2.173–10.532) <0.0001 (15.1)
TJC78	1.113 (0.992–1.249) 0.068 (3.3)	1.178 (11.039–1.336) 0.010 (6.6)		1.261 (1.062–1.497) 0.008 (7.0)	1.058 (0.984–1.137) 0.127 (2.3)	1.203 (1.070–1.352) 0.002 (9.5)		Not retained
CD8 T cells	0.952 (0.917–0.988) 0.009 (6.8)	Not included in the model	0.911 (0.867–0.957) <0.0001 (13.7)	0.908 (0.859–0.959) <0.001 (12.0)	0.960 (0.932–0.989) 0.006 (7.4)	Not included in the model	0.943 (0.905–0.982) 0.005 (7.9)	0.950 (0.911–0.991) 0.018 (5.6)
NK cells CD56^bright^	0.485 (0.178–1.317) 0.156 (2.0)		0.155 (0.038–0.631) 0.009 (6.8)	0.143 (0.027–0.751) 0.022 (5.3)	0.649 (0.294–1.434) 0.285 (1.1)		Not retained	Not retained
NK cells CD56^diml^	1.052 (0.987–1.120) 0.117 (2.5)		Not retained	Not retained	1.047 (1.005–1.090) 0.028 (4.8)		Not retained	1.065 (0.999–1.136) 0.054 (3.7)
Naïve CD4 cells^a^	0.956 (0.934–0.978) <0.0001 (14.8)		0.899 (0.867–0.932) <0.0001 (32.6)	0.892 (0.855–0.930) <0.0001 (28.8)	0.973 (0.958–0.989) <0.001 (11.1)		0.931 (0.906–0.956) <0.0001 (25.8)	0.954 (0.926–0.984) 0.003 (9.1)
IRC CD4 cells	1.245 (1.098– 1.411) <0.0001 (11.7)		Not retained	Not retained	1.055 (1.021–1.090) 0.001 (10.2)		Not retained	1.113 (1.000–1.238) 0.049 (3.9)
Treg CD4 cells^a^	0.651 (0.554–0.765) <0.0001 (27.0)		0.518 (0.416–0.646) <0.0001 (34.2)	0.489 (0.374–0.630) <0.0001 (30.4)	0.765 (0.689–0.851) <0.0001 (24.6)		0.638 (0.544–0.747) <0.0001 (30.8)	0.656 (0.554–0.776) <0.0001 (24.1)
Regulatory B cells	1.164 (1.021–1.325) 0.021 (5.3)		1.253 (1.061–1.479) 0.008 (7.0)	1.205 (1.1001–1.451) 0.049 (3.9)	1.123 (1.024–1.232) 0.013 (6.1)		1.210 (1.053–1.390) 0.007 (7.1)	Not retained
Plasma blasts	1.119 (0.873–1.432) 0.375 (0.8)		1.326 (0.962–1.826) 0.085 (2.9)	1.303 (0.979–1.735) 0.070 (3.3)	1.066 (0.927–1.226) 0.367 (0.8)		Not retained	Not retained
Accuracy (%)	Not applicable	70.0%	77.6%	85.7%	Not applicable			
AUROC (95%Cl) *P*-*value*		0.744 (0.678–0.810) *P* < 0.0001	0.862 (0.814–0.910) *P* < 0.0001	0.911 (0.871–0.951) <0.0001	Not applicable	0.702 (0.697–0.704) *P* < 0.0001	0.773 (0.756–0.760) *P* < 0.0001	0.794 (0.785–0.791) *P* < 0.0001
Sensitivity (%) (95%Cl)		55.9 (55–66)	74.2 (64–83)	83.9 (75–91)	not applicable			
Specificity (%) (95%Cl)		81.2 (73–87)	80 (72–87)	80.3 (72–87)				
PPV (%) (95%Cl)		70 (61–78)	75 (67–83)	77.2 (70–83)				
NPV (%) (95%Cl)		70 (64–75)	80 (73–85)	86.2 (80–91)				
Nagelkerke R square		25%	50%	62%	Not applicable	20%	32%	42%
Hosmer& Lemeshow		0.853	0.912	0.287		Not applicable		
Bias-corrected Somers D_X_y		Not performed		0.458		Not performed		0.533

a Normalised frequency.

AUC: area under the roc curve; EMS: early morning stiffness; Exp-: expanded; HLA (SE): human leucocyte antigen (shared epitope); HR: hazard ratio; IRC: inflammatory-related cells; NK: natural killer; NKT: natural killer-T; NPV: negative predictive value; OR: odds ratio; PPV: positive predictive value; TJC: tender joint count; Treg: regulatory T cells.

**Figure 3.**
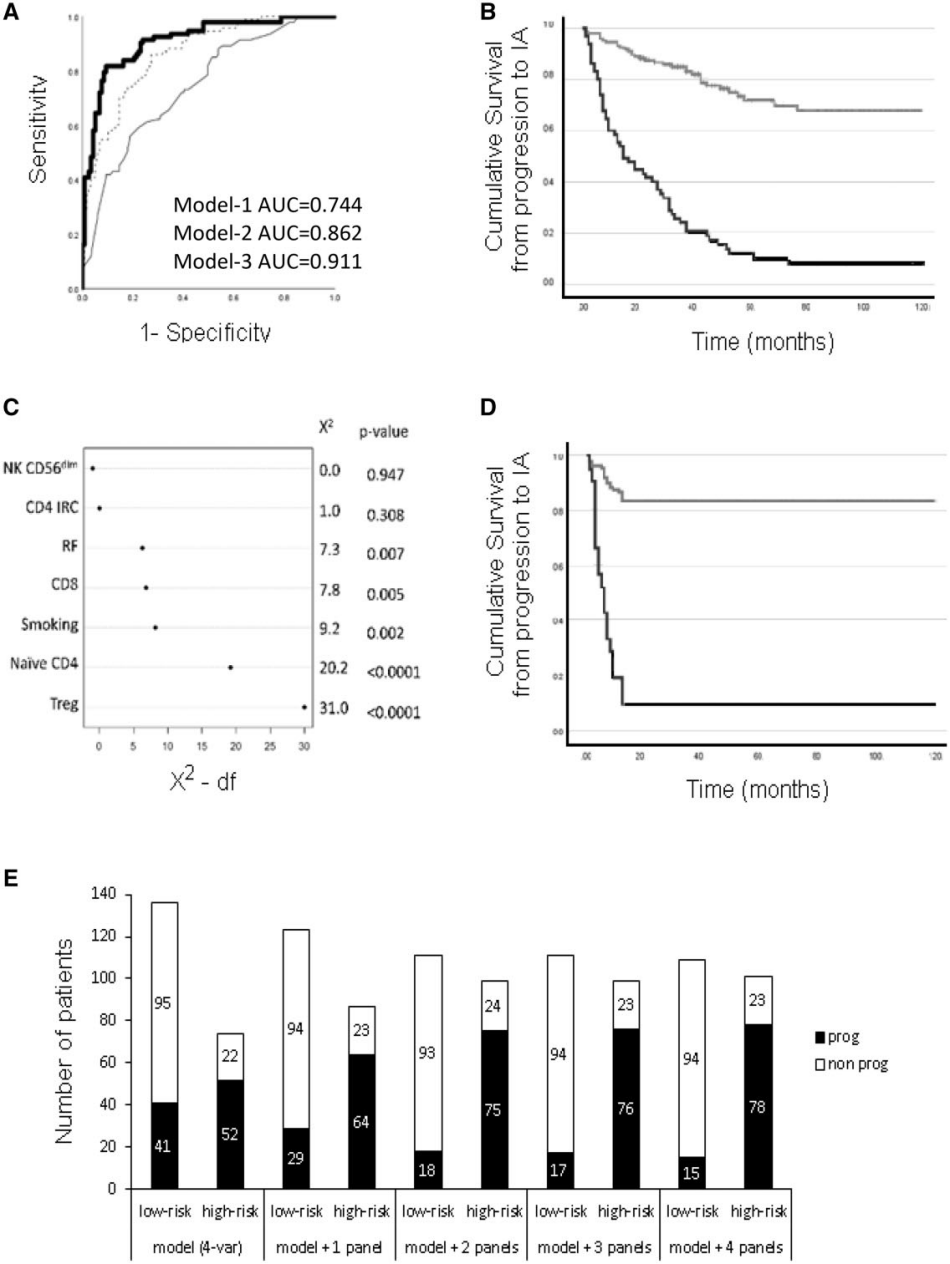
Performances of the models. (**A**) AUROC graphical representation of the logistic regression models. Binary logistic regression models of the occurrence of progression to inflammatory arthritis (IA) were constructed using Model 1 (Clinical data only) for 10 parameters (thin line), Model 2 (flow-data only) for 18 subsets (dotted line) and Model 3 (clinical + flow data, thick line). Model 1 (AUC = 0.744 95%CI 0.678–0.810) was inferior to Model 2 (AUC = 0.862, 0.814–0.910) and Model 3 still showed added value (AUC = 0.911, 0.871–0.951). (**B**) Survival curve based on classification using Model 3. Survival plot analysis was performed after patients were dichotomised for high-risk (black line, *n* = 65/210) and low-risk (grey line, *n* = 145/210) based on individual probability (>0.80%) calculated from the logistic regression. (**C**) Variables contribution to Model 6: This showed the relative importance order of the predictors in the model with Treg as the most discriminating biomarker for rapid progression followed by naiveCD4, smoking, CD8, RF, IRC-CD4 and finally NK-CD56^dim^ cells. (**D**) Survival curve based on the Cox regression for rapid progression. Survival plot analysis was performed after patients were dichotomised for high-risk (black line, *n* = 22/158) and low-risk (grey line, *n* = 136/158) based on individual hazard (>2) calculated from the Cox regression. (**E**) Overall performance of the prediction model using 1 to 4 flow cytometry panels. Individual participants’ probability for progression was dichotomised into high/low-risk groups (based on 80% specificity) in five logistic regression models including the demographic/clinical data only first and then, sequentially adding data from 1, 2, 3 and then 4 flow-cytometry panels. Numbers of patients in both risk groups are displayed against the number of progressors (black bars) and non-progressors (open bar)

Model-1 selected four parameters in a stepwise construction, starting with RF, then sequentially adding HLA-SE, TJC78 and smoking. This model accurately predicted 70% of cases, with SEN/SPE = 56%/81.2% and a good NPV/PPV = 70%, with an AUC = 0.744 (Fig. 3A). However, only 25% of the variance for predicting progression was accounted for (Nagelkerke R-square) and individual variable contribution was 17% for RF and <7% for the other three (individual Wald score).

Model-2 excluded many subsets and retained only five: Treg, naive CD4+T cells, CD8+T cells, B-reg and finally NK-CD56^bright^ cells with an overall accuracy = 77.6%. An additional step adding plasmablasts (not independently predictive) still increased accuracy (+7.6%) with an improvement of AUC = 0.862 (+12%) compared with model-1. Model-2 accounted for 50% of the variance with Treg (34%), naive CD4+T cells (32%) and CD8 (14%) contributing with the highest and other LS EACH for >6%.

Combining both datasets, Model-3 showed added value (+8.1% accuracy > model-2) using 10 steps selecting Treg and naive CD4+T cells, then smoking/RF, followed by CD8+T cells, TJC78/HLA-SE and finally NK- cells-CD56^bright^, plasmablasts and B-reg. Model 3 had final accuracy = 85.7% and AUC = 0.911 (+ 4.9% compared with model-2), altogether accounting for 62% of the variance with again Treg (30%), naive CD4+T cells (29%), CD8 (12%) and RF (10%) contributing the most with <7% for all other variables.

We further applied internal validation to model-3 to correct for optimism using a bootstrapping technique (500 permutations), resulting in an optimised Dxy-value = 0.458. We ran a calibration and observed no overfitting of the bootstrapping technique (slopes was close to 1; Supplementary Fig. S5, available at *Rheumatology* online).

The high PPV/NPV of model-3 (77%/86%) suggests that it is possible to predict individuals who are likely to progress while identifying those who have low risk and may be monitored less often (even discharged). In this cohort, using dichotomisation based on the probability to progress calculated for each patient in model-3, 65/210 could be deemed high-risk (Fig. 3B, i.e. probability >80%) and all but six progressed (91% accurate). Alternatively, 78/210 cases were low-risk (i.e. probability <20%) and only six (7.7%) progressed over 10 years.

### Modelling rapid progression to IA

Time to progression is widely distributed in this cohort ranging from 1–120 months. Different LS associations may therefore be involved at different stages of the progression, and some may be more predictive of the onset of IA symptoms than others. A total of 41/93 (44%) of the progressors did so rapidly and we re-analysed these progressors using a Cox regression.

The assumption or proportional hazard was verified for all variables in model 6 (Supplementary Fig. S4, available at *Rheumatology* online). Cox regression models were constructed using the same forward approach (Table 3). Un-adjusted hazard ratio (HR) for time to progression was significant for four clinical variables (smokers/HLA-SE/RF/EMS, CRP showing a trend) and for six LS (Treg/naive CD4+T cells/CD4-IRC/CD8+T cells/NK-CD56^dim^/B-reg).

Model-4 (demographic/clinical only) retained three variables sequentially, starting with RF, TJC78, and smokers with an AUC = 0.702. Model-5 (LS only) used 4 steps and retained Treg, naive CD4+T cells, CD8+T cells and B-reg, with an improved AUC = 0.773. Model-6 (combined datasets) used seven steps with Treg, RF and CD4-IRC then smokers, NK-CD56^dim^, naive CD4+T cells and finally CD8 T cells, with an AUC = 0.794, showing significant discrimination index X^2^ for the included variables, ranging from 31.0 for Treg to 0.00 for NK-CD56^dim^ cells (Fig. 3C). The bootstrapping approach showed that optimism corrected Dxy = 0.533. The calibration also showed no overfitting of the bootstrapping technique (Supplementary Fig. S5, available at *Rheumatology* online).

Being able to identify individuals at high risk of rapid progression would allow for the design of a clinical trial aiming at the prevention of progression within a short trial duration of only 12 months. In this group, 22 participants were dichotomised based on a high-hazard (individual X-beta score value > 2) for rapid progression in model-6, and 20 (91%) progressed (Fig. 3D).

### Applicability for daily practice

Flow cytometry is routinely used in Leeds and worldwide. We therefore evaluated the gain in terms of accurate stratification of adding LS panels (cost/time/technology) over only using demographic/clinical data. Individual participants’ probability of progression was dichotomised (based on a cut-off at 80% specificity) into high/low risk groups, for the demographic/clinical data then sequentially adding 1–4 flow-cytometry panels (Supplementary Table S3, available at *Rheumatology* online).

The stratification showed 70% accuracy for the reference model (Fig. 3E). Adding the Treg panel increased accuracy to 75.2%. Adding the naive CD4+T cells panel showed further accuracy = 80.4% while adding CD8+/NK-cells (lineage panel) and then B-reg (B-cell panel) only achieved a marginal improvement of accuracy (to 81%/81.5%, respectively). The AUC, however, was still improving with every incremental step and all four panels added value.

A similar analysis for imminent progression suggested that only three panels would be needed while the performances clearly improved with the three steps (Supplementary Table S4, available at *Rheumatology* online).

## Discussion

Our study demonstrated the value of extensive phenotyping of LS to predict progression to IA in ACPA+ at-risk individuals. The clustering analysis suggested that progressors are associated with specific LS profiles, cluster–III dominated by high CD4-IRC and cluster-II by high plasmablasts/Treg. Non-progressors (cluster-I) were characterised by high Treg/CD4-naive and low plasmablasts/CD4-IRC. These profiles were not associated with any particular demographic/clinical data, suggesting that they are independently regulated from any genetic/environmental or inflammatory events. We, therefore, confirmed previous data for CD4+T-cell subsets and further identified LS associated with progression, providing clues to the identity of cells (CD8/NK/B-cells) involved in the events triggering or associated with progression. Importantly, different subsets were retained in the modelling for overall compared with imminent progression, suggesting a time frame for different biological roles/triggers. Overall, our data confirm that wider LS dysregulation precedes the development of clinical synovitis, while also providing increased accuracy over previous models with only CD4+T-cell subsets.

Our findings showed an increase in circulating B-reg frequencies paralleling a reduction in Treg, suggesting an additional role for regulatory mechanisms before the onset of clinical synovitis. Functionally defective B-regs have been associated with homing to synovitis [24] while other studies have associated inflammation with the expansion of B-regs [25, 26]. In new-onset RA, the failure of B-reg to maintain a functionally suppressive Treg population was demonstrated [24, 27]. It is therefore conceivable that a decrease in Treg (loss of tolerance), paralleled with an increase in B-regs (subclinical inflammation) are both associated with progression to IA. On the other hand, our findings do not exclude that B-regs may be functionally defective [28, 29] or were excluded from the synovium (i.e. circulating), limiting their ability to perform their role locally. Future work will be needed to determine which hypothesis may be correct.

Consistent with our original work [15, 17], a reduction in circulating naive and Treg CD4+T cells frequencies and an increase in IRC-CD4 predates the development of IA (AUC = 0.790, *n* = 102). Since this original work, a standardised normalisation procedure for the naive/memory and Treg subsets was established [15, 17], allowing for analysis of different phase-specific outcomes across the IAC using continuous data. Applying this to this group of 210 patients provided further validation of the original three CD4+T-cell subset model using the same logistic regression approach based on entering all variables in the model (enter method); however, it suggested that IRC-CD4 were no longer independently contributing to the prediction in this larger group (OR = 1.104, *P* = 0.144). Modelling using a forward method allowing for the best predictors only (data not shown, accuracy = 78.6%, AUC = 0.880) confirmed that IRC were less predictive of the overall progression over 10 years, while still highly associated with rapid progression over 12 months, as also observed in the clustering (Fig. 2) where most of the rapid progressors [28/41 (68%)] were in Cluster III defined by higher CD4+IRC.

We have associated many defects in the naive CD4+T-cell subset with early RA pathogenesis, notably in relation to a decline in thymic activity, aberrant signalling and aberrant proliferation reducing their TREC (T-cell receptor excision circle) content by >50% (1–2 cell cycle) [13], impaired IL7 responsiveness [30] and recently an IL6-driven network of epigenetic modifications suggesting the development of a subpopulation expressing more pro-inflammatory cytokines and closely resembling IRCs [31, 32]. In addition, naive CD4+T-cell loss was also shown to directly result from the differentiation of naive cells into IRC [13], a process driven by inflammation directly related to measures of inflammation [13] with a central role for IL6 in driving such changes and loss of IL6R expression as a result of its signaling [31]. Furthermore, IRCs are persisting when inflammation is subclinical due to reduced expression of pro-apoptotic genes [32] and are associated with the occurrence of flares in patients in synthetic-DMARDs-induced remission [33]. Most importantly, IRCs remained naive to an antigen challenge (hence expressing CD28) [13, 32], and were shown to be recent progeny of naive cells with a high content of T-cell receptor excision circles (TREC) [13]. As such, they are not to be confused with terminally differentiated T-effector memory T cells (TEMRA) re-expressing CD45RA, with controversies about high/low levels of expression of CD45RO and expression of CCR7 (reported negative or positive), while more consistently lacking CD62L, CD27 and CD28 expression, and also differences between CD4+ and CD8+ T cells [34–37] which are antigen-experienced (with low TREC content). Although, the 5-colour panel used here does not include all the markers that would definitely differentiate all the various subsets of memory T cells (notably TEMRA from IRCs), this does not alter the biomarker value of the IRC phenotype identified in this study. Indeed, here, we observed that IRC-CD4 enables segregation of a particular cluster of patients, the majority being rapid progressors, 28 of the 41 (68%) progressors being in Cluster-III. This further supports the hypothesis that imminent progression towards IA may be driven by an event involving or resulting from subclinical inflammation, driving or being associated with the differentiation of naive CD4+T cells into IRC as previously hypothesized [13, 17, 31]. Other CD4+T-cell subsets are relevant to RA pathology, notably Th17 cells and Tfh cells [38, 39]. However, it would have been very limiting to include panels for these in 2017–19 having started this work long before. Alternatively, the addition of a test for the enumeration of Th17 cells using a DNA-methylation specific qPCR [40] is, however, possible and should prove informative using stored whole blood.

Although CD8+T cells share some of the pathways genetically associated with CD4+T cells in RA [41], they have not been reported for their potential biomarker value to our knowledge, with one report presenting an association with arthralgia [42]. In preclinical IA, reports, however, suggest that CD8+T cells make up ∼40% of the total T cells infiltrating the synovium [43, 44]. Following prolonged/chronic exposure to infectious agents, alterations in homing molecules expressed by CD8+T cells occur [45], resulting in enhanced/altered migration. Here, we indeed demonstrated a reduced frequency of circulating CD8+T cells, predictive of progression that could be reflecting migration/accumulation into the joint. Alternatively, this reduction could reflect a contraction of the CD8+T-cell pool following an infection (post activation cell death), suggesting a role for subclinical infections as additional environmental risk for RA, as proposed in the past [46]. Furthermore, cell death causes the release of inflammatory mediators that might also serve as triggers of cascades of events, particularly netosis [47]. In our study, NK-cells CD56^dim^ were also predictive of progression suggesting that dysregulation of NK cells may have a significant biological role, further fuelling an inflammatory cascade leading to disease progression. Several studies have indeed supported a role for NK cells in RA pathology [48, 49] and at the onset of disease in ACPA+ RA and in pre-RA (arthralgia), although this observation was not directly related to progression [50].

The clinical predictors in the models developed in this study were slightly at variance with those from a previously published model [6] probably because different patients and parameters were used (notably 78-TJC rather than hands small TJC and importantly, ACPA-positivity based on a CCP2 test). Model-1 notably only explains 25% of the variance in predicting IA. Modelling using combined data (model-3) showed that six LS and the same four clinical parameters had superior value over model-2 and clearly over model-1 (+15.7% accuracy). Nonetheless, only 62% of the variance can be explained by model-3, leaving room for additional makers to be added, possibly using imaging and cytokines or epigenetic modification as recently evidenced in early RA [31]. Further comparison with other models [6, 7] is difficult as they did not use the same statistical approach (also not providing AUC) but our model 3 showed high performance (AUC = 0.911) for the overall prediction, while rapid progression is slightly less good but still high (AUC = 0.794). On the other hand, we observed similar findings in RF+ only patients (4/13 progressors, data not shown) which also confer risk for IA development in patients with arthralgia [19]. This was verified for naive, Treg, IRC and CD8 but not for NK and the B-cell subsets (possibly due to small numbers). This nonetheless suggests potential predictive value for these LS across at least two risk-related autoantibodies. Further work would be needed to evaluate these with respect to other risk factors [4, 5]; however, here we already showed higher value for LS than for known risk factors such as genetic (HLA-DR SE) and/or lifestyle (smoking).

Another major clinical benefit of being able to predict rapid progression would be for patients to access treatment at the critical very early IA/RA stage (i.e. 1–2 weeks of detectable synovitis) and to assess whether this can affect the long-term outcome/prognostic of these patients compared with routine early arthritis referrals (up to 2 years symptom duration). These are all studies currently ongoing in Leeds which we hope to report in the future. Although few RA prevention trials have been reported (many being in progress/planned), it remains unclear whether IA development can be prevented in at-risk individuals and which drug/regimen would be most appropriate. Recent trials nonetheless suggest this may be possible: the APIPRRA study (ISRCTN-46017566 using abatacept [51]) showed a sustained preventive effect at 2 years (data presented at EWRR and EULAR 2023); the PRAIRI study (using rituximab) [52] also showed delayed progression. Our data add valuable information that could contribute to risk stratification (as well as understanding of the biology of the at-risk phase), but do not in themselves justify treatment for the time being. Different LS being indicative of the imminence of IA, our data may therefore find their best utility in selecting possible interventions targeting these cells/subsets/events, supporting personalised clinical decision making, and guiding the selection of patients best suited for such preventive intervention.

The limitations of our work include the relatively low number of subjects with all flow panels and the selection of ACPA+ participants with a highly specific CCP-2 test. We also recognise the limitation of the statistical modelling approaches and performed the optimism correction to account for this in models 3 and 6, while the model with only the three CD4+T-cell subset reproduced previous data [15, 17]. A second (external) cohort would be critical to fully validate these findings, the main hurdle in any biomarker research programme being that it can only be replicated if the selection criteria of the study population are the same. There are many cohorts of at-risk individuals worldwide [5] but they all use different criteria to define individuals at risk of RA, preventing the generalisation of any findings as previously discussed [5]. The data presented here nonetheless replicate previous models, while the final model using more subsets showed clear improvement.

On the practical side, although we transferred this technology to NHS services (back in 2013, based on the use of fresh whole blood samples), currently these specific panels are only available in Leeds, while the technology itself is used worldwide and can provide data for clinical use in about 3–5 h. Protocol that would allow for frozen blood samples (using SmartTubes^TM^) [13] could facilitate the use of these panels in a single flow centre (i.e. for retrospective analysis of trials samples for example). Alternatively, the technology can be replicated as recently shown in a collaborative work between Leeds and France [53]. A careful planning of the number of antibodies/panels could be rationalised to suit local technical flow machine capacity (using SSC/CD4 gating for example). Novel technical development such as the use of dry tubes, whereby antibodies are pre-coated on flow plastic tubes would also considerably help reduce procedure time (no pipetting) and increase adherence to SOP.

In conclusion, our study suggests that LS homeostasis is dysregulated at the early stage of the RA disease continuum before clinical synovitis occurs. We have demonstrated the additional predictive value of CD8+ T cells B-reg and NK-cells besides the previously established CD4+T-cell subsets. We demonstrated that perturbations in different subsets are associated with progression to arthritis, including rapid progression within 12 months, suggesting that additional time-dependent cell-based events are necessary for the progression to IA, while the development of systemic autoimmunity is not sufficient alone. In addition, these panels are simple to perform routinely offering new tools to manage and stratify the risk of developing IA in ACPA+ at-risk individuals.

## Supplementary Material

kead466_Supplementary_Data

## Data Availability

The raw data underlying this article cannot be shared publicly to protect the privacy of individuals who participated in the study. Anonymised data could be shared on reasonable request to the corresponding author.
