# Supplementary material for: Lymphocyte subset phenotyping for the prediction of progression to inflammatory arthritis in anti-citrullinated-peptide antibody-positive at-risk individuals
Source: Rheumatology (Oxford). 2023 Sep 7;63(6):1720–32. doi: 10.1093/rheumatology/kead466 (PMC11147546; doi:10.1093/rheumatology/kead466)
Supplement: kead466_Supplementary_Data [file kead466_supplementary_data.docx]

**Supplementary material**

**Lymphocyte subsets phenotyping for the prediction of progression to Inflammatory Arthritis in ACPA+ at-risk individuals.**

**Authors** Innocent Anioke^1,$^, Laurence Duquenne^1,2^, Rekha Parmar^1^, Kulveer Mankia^1,2^, Farag Shuweihdi^3^, Paul Emery^1,2^, Frederique Ponchel*^1^

IA, ORCID: orcid.org/0000-0001-7600-4823

LD, ORCID: orcid.org/0000-0001-7631-0986

RP, ORCID: orcid.org/0000-0002-8002-6440

KM, ORCID: orcid.org/ 0000-0002-7945-6582

FS, ORCID: orcid.org/0000-0003-1199-2992

PE, [ORCID: orcid.org/0000-0002-7429-8482](http://orcid.org/0000-0002-7429-8482)

FP ORCID: orcid.org/0000-0002-3969-7701

**Address**

1. Leeds Institute of Rheumatic and Musculoskeletal Medicine, University of Leeds, Leeds, UK

2. NIHR Leeds Biomedical Research Centre, Leeds Teaching Hospitals NHS Trust, Leeds, UK

3. Leeds Institute of Health Sciences. University of Leeds, School of Medicine.

^$^ Additional affiliation: Department of Medical Laboratory Sciences, University of Nigeria, Nigeria.

***Corresponding author:** Frederique Ponchel

**`**  Leeds Institute of Rheumatic and Musculoskeletal Medicine,

St James Hospital, CSB, room 5.25

University of Leeds,

Leeds, UK., Leeds LS9 7TF,

UK.

**Flow cytometry data retrieval, analysis, and QA/QC**

Blood samples at inclusion in the study were sent from clinics at Chapel-Allerton Hospital, to the NHS immunology services at the St James hospital using NHS transport services, as previously detailed(15). Blood LS data were acquired within a few hours of the samples arriving at the NHS immunology lab, with some exceptional delays on some occasion (fully documented).

All selected patients had data for the CD4+T-cells panel (naïve, IRC) and the Lymphocyte count panel. For the Treg panel, a shortage of 1 antibody (FoxP3) led to missing data over a few weeks (n=20/201). For the CD8 panel, we had an issue with poor separation between markers in samples for which processing was delayed by transport. The B-cell panel was the most limiting for inclusion as it was introduced late (2015), hence limiting the cohort to 210 patients amongst the >450 ACPA+ participants recruited. During the analysis of the 5 panels, a few more issues were encountered necessitating data to be excluded due to poor-quality of samples mainly related to delay in transport affecting more than the CD8+T-cells panel, faulty acquisition due to non-adherence to sample preparation protocols or problems encountered over data acquisition (for example the flow machine breaking down). This led to 210 cases only being selected.

The raw data (FCS files) were made available for use in the current study as electronic files. Flow-cytometry analysis for the quantification of blood cell subsets included panels for lineage count, B-cell subsets; CD4+T-cell subsets; CD8+T-cell subsets; and CD4+Treg. Antibody clones and staining protocols used are described in detail in a previous publication (15). Flow cytometry analysis was performed on an LSRII cytometer (Beckon Dikson, Biosciences and FACS-DIVA software). The gating strategies were previously described (15,20) and further illustrated in Sup Figure 1. Blood subsets frequencies were reported as a percentage of the parental population.

Age-relationship for specific cell subsets were previously described in healthy controls over 6 decades of age using 120 healthy controls(15). A normalisation was applied as previously detailed, resulting in frequencies above or below expected values(20). The Breg cell frequencies had not previously been correlated with age (15). However, with the addition of more healthy controls (n=30), the frequency of Breg was still less likely associated with age (rho < 0.600) as such age normalisation was not applied to the subset in this study.

**
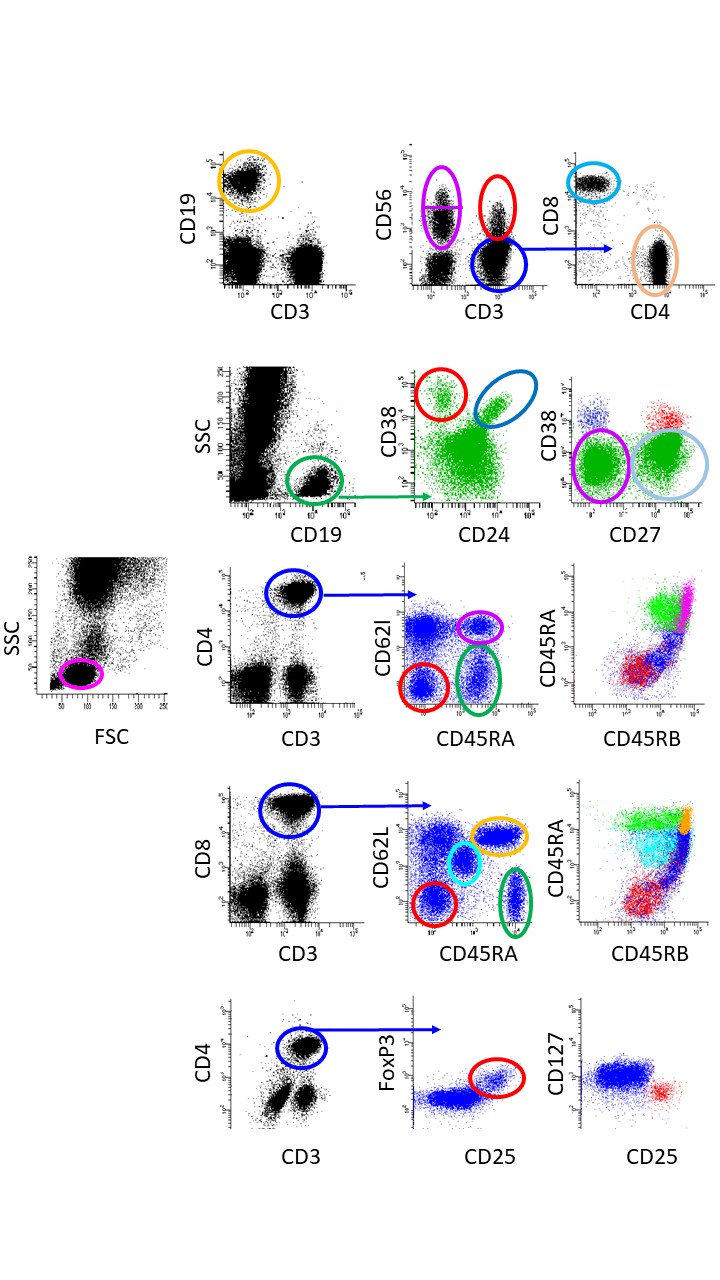
**

**Figure S1**. **Gating** **strategies for 18 LS quantification**.

A lymphocyte gate [pink] was applied to all panels (except for the B-cell subsets).

**Top row: Lymphocyte linages** were quantified using 5 lineages markers including [orange] B-cells CD19+, [purple bottom] NK cells CD56^dim^, [purple top] NK cells CD56^bright^, [red] NKT cells CD56+CD3+, [light blue] CD8+T-cells and [light brown] CD4+ T-cells based on a [dark blue]T-cells gate (CD3+ / CD56- cells).

**Second row:** B-cells [green] were gated based on CD19 expression and side scatter. B-cell subsets were gated based on the expression of CD24, CD27, and CD38 for [purple] naïve cells (CD27-/CD38+), [cyan] memory cells (CD27+CD38+), [red] plasmablasts (CD27+/CD38^high^) and CD24^high^/CD38^high^ was used to define regulatory B-cells [blue] (B-reg).

**3^rd^ row: CD4+T-cells** [blue] gate : CD4+T-cells subsets (left plot gated on a CD3+ cells) were gated based on the expression of CD45RA and CD62L for [purple] naïve cells (CD45RA+/CD62L+), [green] inflammation related cells IRC (CD45RA+/CD62L-), and [red] memory cells (CD45RA-/CD62L-). CD45RB was used to refine the naïve gate [coloured in purple] as CD45RB^bright^ and the IRC [coloured in green] as CD45RB^intermediate^, while in the memory cells gate [coloured in red] CD45RB expression was lower.

**4^th^ row: CD8+T-cells** [blue] gate: CD8+T-cells subsets (left plot gated on a CD3+/CD8+ cells) were gated for [orange] naïve cells (CD45RA+/CD62L+/CD45RB^high^), [green] IRCs (CD45RA+/CD62L-/CD45RB^intermediate^ to CD45RB^low^), and [red] memory cells (CD45RA-/CD62L-/CD45RB^low^). An expended population of CD8 cells [cyan] with intermediate levels of expression of all 3 markers CD45RA, CD62L, and CD45RB was clearly seen in some participants (as previously described [19]) but not in most. Of note, the cyan gate on the right panel for the expanded subset (CD45RB^internmediate /low^/CD45RA^low^) may not exclusively include the expended CD8 population.

**Bottom row: Regulatory CD4+T-cells:** CD4+T-cells [blue] were gated for [red] regulatory T-cells (Treg) based on the high expression of CD25 and FoxP3. Treg cells were then looked at on a CD25/CD127 plot to adjust the position of the Treg gate [coloured in red], so that no CD25^low^ cells were included, and to confirm the lower expression of CD127.

**Details of the follow-up duration in progressors and non-progressors**

The length of follow-up was very wide, 120 months in total with a median follow-up duration was 34 months +/- 31 months SD overall. The shortest time to progression was 1 month while the longest was 120 months. The shortest follow-up considered in non-progressors was 12 months. The cohort was spitted between imminent progressors and then 4 time groups of delayed progression by 1 year, 2 years, 3-5 years and more than 5 years, as displayed in the histogram below, and showing the number of progressors and that of non-progressors with the same duration of follow-up.

**Figure S2: Follow-up duration and progression**


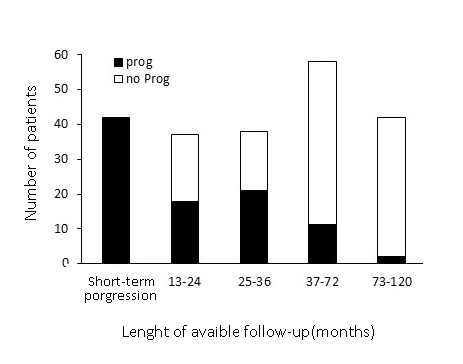


**Figure S3: Unsupervised hieratical clustering of 18 subsets in individuals at-risk of developing IA (n=210).**

**
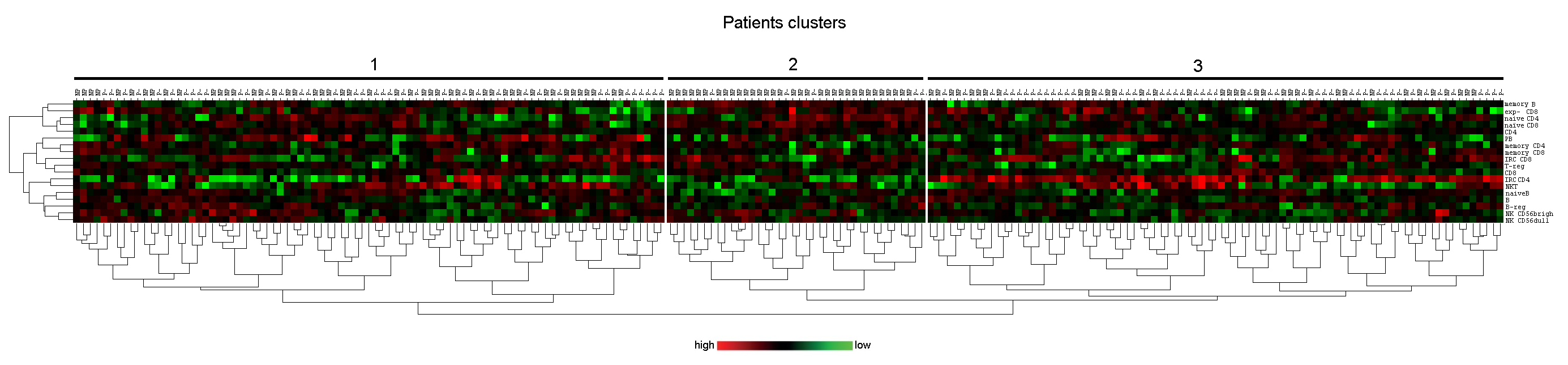
**

**Figure S3:** An unsupervised hierarchical clustering algorithm was applied to log transformed frequencies for 8 LS (n=210) and results are displayed as a heat-map of data (red being the highest and green the lowest frequency observed for each LS). This clustering algorithm builds relationships between LS frequencies based on Spearman rank correlations, and segregates patients into 3 clusters (annotated I, 2, and 3), based on 3 main groups of LS. The 1^st^ group included 5 subsets which frequency reduced with age in health (naïve CD4/CD8 T-cells and memory B-cells) as well as CD4+T-cells and the expanded-CD8 subset. The 2^nd^ group clustered 6 subsets including those increasing with age (memory CD4/CD8 T-cells, Treg) as well as CD8+T-cells, CD8-IRC, and PBs. The 3^rd^ group gathered 7 subsets; B-cells, naïve B-cells, and Breg, as well as NK (CD56 bright and dim), NKT-cells, and CD4-IRC. Therefore, each group of LS included at least one of the subsets individually highly associated with progression. Cluster of patients showed significantly different proportions of progressors (p<0.0001) with 39/90 (45%) progressors in the cluster-1, depleted with 5/38 (13%) in cluster-2, and enriched in cluster-3 with 49/82 (60%). NP= Non-progressors, P= progressors.

**Table S1: Univariate performance index of the 18 subsets in relation to association with predicting progression to IA**

|  | SENSITIVITY  (95%Cl) | SPECIFICITY  (95%Cl) | PPV  (95%Cl) | NPV  (95%Cl) | overall % accuracy  (95%Cl) |
| --- | --- | --- | --- | --- | --- |
| CD4  T-cells | 20.51  (13.61 to 28.97) | 80.12  (73.34 to 85.82) | 41.38  (30.68 to 52.95) | 59.57  (56.68 to 62.38) | 55.90  (49.96 to 61.72) |
| CD8  T-cells | 32.48  (24.11 to 41.76) | 80.12  (73.34 to 85.82) | 52.78  (42.87 to 62.47) | 63.43  (59.97 to 66.75) | 60.76  (54.86 to 66.44) |
| B cells | 21.37  (14.33 to 29.91) | 80.70  (73.98 to 86.33) | 43.10  (32.28 to 54.63) | 60.00  (57.10 to 62.83) | 56.60  (50.66 to 62.40) |
| NK cells CD56^bright^ | 23.93  (16.53 to 32.70) | 80.12  (73.34 to 85.82) | 45.16  (34.62 to 56.15) | 60.62  (57.57 to 63.59) | 57.29  (51.35 to 63.08) |
| NK cells  CD56^dim^ | 30.77  (22.57 to 39.97) | 80.12  (73.34 to 85.82) | 51.43  (41.38 to 61.36) | 62.84  (59.47 to 66.10) | 60.07  (54.16 to 65.77) |
| NKT  cells | 26.50  (18.77 to 35.45) | 80.12  (73.34 to 85.82) | 47.69  (37.32 to 58.27) | 61.43  (58.27 to 64.51) | 58.33  (52.40 to 64.09) |
| Naïve  CD4 cells ^$^ | 39.32  (30.41 to 48.77) | 80.12  (73.34 to 85.82) | 57.50  (48.16 to 66.33) | 65.87  (62.09 to 69.45) | 63.54  (57.69 to 69.11) |
| Memory  CD4 cells^$^ | 25.64  (18.02 to 34.54) | 80.12  (73.34 to 85.82) | 46.88  (36.44 to 57.59) | 61.16  (58.03 to 64.20) | 57.99  (52.05 to 63.75) |
| IRC  CD4 cells | 39.32  (30.41 to 48.77) | 80.12  (73.34 to 85.82) | 57.50  (48.16 to 66.33) | 65.87  (62.09 to 69.45) | 63.54  (57.69 to 69.11) |
| Treg  CD4 cells^$^ | 59.83  (50.36 to 68.78) | 80.12  (73.34 to 85.82) | 67.31  (59.55 to 74.22) | 74.46  (69.77 to 78.64) | 71.88  (66.30 to 76.99) |
| Naïve  CD8 cells ^$^ | 23.93  (16.53 to 32.70) | 80.12  (73.34 to 85.82) | 45.16  (34.62 to 56.15) | 60.62  (57.57 to 63.59) | 57.29  (51.35 to 63.08) |
| Memory  CD8 cells ^$^ | 32.48  (24.11 to 41.76) | 80.12  (73.34 to 85.82) | 52.78  (42.87 to 62.47) | 63.43  (59.97 to 66.75) | 60.76  (54.86 to 66.44) |
| Exp-memory like CD8 cells | 30.77  (22.57 to 39.97) | 78.95(72.07 to 84.80) | 50.00  (40.19 to 59.81) | 78.95  (72.07 to 84.80) | 59.38  (53.46 to 65.10) |
| IRC  CD8 cells | 23.08  (15.79 to 31.77) | 80.12  (73.34 to 85.82) | 44.26  (33.68 to 55.40) | 60.35  (57.35 to 63.28) | 56.94  (51.01 to 62.74) |
| Naïve  B cells ^$^ | 22.22  (15.06 to 30.84) | 80.12  (73.34 to 85.82) | 43.33  (32.71 to 54.61) | 60.09  (57.12 to 62.98) | 56.60  (50.66 to 62.40) |
| Memory  B cells^$^ | 23.93  (16.53 to 32.70 | 79.53  (72.70 to 85.31) | 44.44  (34.05 to 55.35) | 60.44  (57.37 to 63.44) | 56.94  (51.01 to 62.74) |
| Regulatory  B cells | 35.90  (27.24 to 45.29) | 80.12  (73.34 to 85.82) | 55.26  (45.64 to 64.51) | 64.62  (61.01 to 68.08) | 62.15  (56.28 to 67.78) |
| Plasmablasts | 38.46  (29.62 to 47.91) | 81.29  (74.62 to 86.83) | 58.44  (48.84 to 67.44) | 65.88  (62.19 to 69.38) | 63.89  (58.05 to 69.44) |

PPV, positive predictive value; NPV, negative predictive value. CI, Confidence interval**. Table S2: Three CD4+T-cell subsets modelling (n=210): unadjusted and multivariate logistic regression using an enter method as previously reported (15)**

|  | Logistic regression OR (95% CI) p-value | | | |
| --- | --- | --- | --- | --- |
|  | **unadjusted** | **Model-1** | **Model-2** | **Model-3** |
| Smokers  (ever) | 2.520  (1.390-4.571)  0.002 | 2.282  (1.1.187-4.388)  0.013 |  | 4.020  (1.740-9.290)  0.010 |
| HLA-SE  positive | 2.963  (1.651- 5.316)  <0.0001 | 2.527  (1.335-4.782)  0.004 |  | 2.747  (1.256-6.007)  0.011 |
| RF  positive​ | 3.710  (2.087-6.593)  <0.0001 | 3.600  (1.947-6.656)  <0.0001 |  | 3.776  (1.773-8.040)  0.0001 |
| TJC78 | 1.113  (0.992-1.249)  0.068 | 1.178  (11.039-1.336)  0.010 |  | 1.216  (1.030-1.435)  0.021 |
| Naïve  CD4 cells ^$^ | 0.956  (0.934-0.978)  <0.0001 |  | 0.932  (0.905-0.952)  <0.0001 | 0.926  (0.898-0.957)  <0.0001 |
| IRC  CD4 cells | 1.245  (1.098- 1.411)  <0.0001 |  | 1.099  (0.958-1.232)  0.177 | 1.104  (0.955-1.300)  0.144 |
| Treg  CD4 cells^$^ | 0.651  (0.554-0.765) <0.0001 |  | 0.578  (0.476-0.704)  <0.0001 | 0.528  (0.420-0.662)  <0.0001 |
| Accuracy  (%) |  | **69.5%** | **71.9%** | **78.6%** |
| AUROC  (95%Cl)  p-value |  | 0.744  (0.678-0.810) p<0.0001 | 0.807  (0.714-0.865) p<0.0001 | 0.880  (0.831-0.921)  <0.0001 |
| Sensitivity (%) (95%Cl) |  | 55.9  (55-66) | 62.3  (52-72) | 73.1  (63-82) |
| Specificity (%) (95%Cl) |  | 81.2  (73-87) | 74  (65-81) | 82.9  (75-89) |
| PPV (%)  (95%Cl) |  | 70  (61-78) | 63  (55-70) | 77.3  (69-84) |
| NPV (%)  (95%Cl) |  | 70  (64-75) | 73.5  (68-78.5) | 79.5  (73-84) |

HLA (SE), human leucocyte antigen (shared epitope); TJC, tender joint count; RF, rheumatoid factor; Treg, regulatory T-cells; IRC, inflammatory-related cells; ^$^ normalised frequency, OR, odd ratio; AUC area under the roc curve, PPV, positive predictive value; NPV, negative predictive value. CI, Confidence interval.

**Figure S4: Testing the Proportional Hazards Assumption**


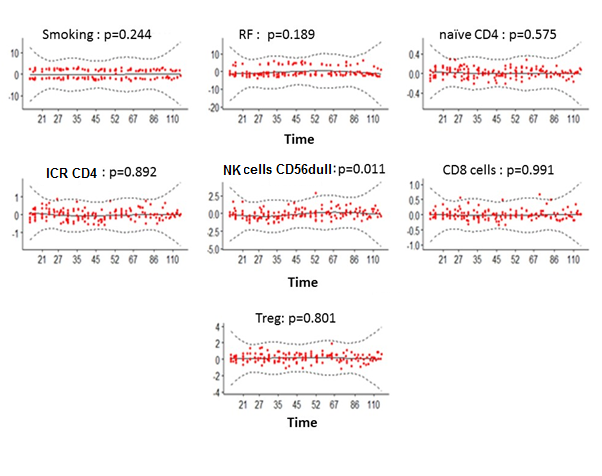


Graphics show that the proportional Hazards Assumption was satisfactory: global Schoenfield test P=0.047. The smooth curve was fairly level across the time horizon here, as opposed to substantially increasing or decreasing in level as time passes. So, the proportional Hazards Assumption was satisfied. Individual Schoenfield test P-values are indicated on each plot.

Although focussed on prediction (based on the ability to discriminate data into classes), our models were validated for calibration, which allows us to verify the closeness of model probability to the underlying probability of the population studied (10.1186/s12916-019-1466-7). Calibration slopes were compared.

**Figure S5: Calibration analysis for the predictive model**

We performed the calibration analysis and observed no overfitting of the bootstrapping technique as slopes were close to 1 for both the logistic and Cox regression.


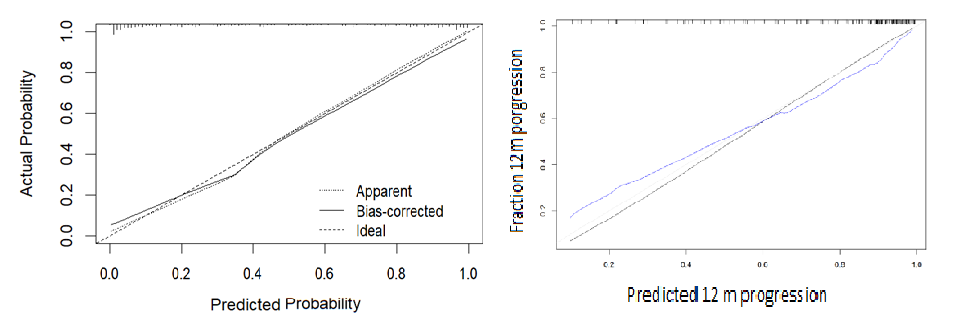


**Table S3: Practical approach to predicting overall progression to IA (n=210)**

| **Logistic regression** | **Logistic regression** | | | | |
| --- | --- | --- | --- | --- | --- |
|  | **Model-ref** | **Model +**  **1 panel** | **Model +**  **2 panels** | **model +**  **3 panels** | **model + 4 panels** |
| **Clinical**  **data** | 4 clinical  variables | 4 clinical variables | 4 clinical  variables | 4 clinical variables | 4 clinical  variables |
| **Flow panel -1**  **Treg** |  | Treg CD4 | Treg CD4 | Treg CD4 | Treg CD4 |
| **Flow panel -2**  **CD4 T cells** |  |  | Naïve CD4 | Naïve CD4 | Naïve CD4 |
| **Flow panel -3**  **lineage** |  |  |  | CD8 T-cells  NK cells CD56^bright^ | CD8 T-cells  NK cells CD56^bright^ |
| **Flow panel -4**  **B-cell** |  |  |  |  | B-reg  PBs |
| **Accuracy**  (95% CI) | 70.0%  (63.31- 76.11) | 75.24%  (68.83 – 80.92) | 80.0%  (73.93 - 85.19) | 81.0%  (74.98 - 86.03) | 81.9%  (76.02 - 86.87) |
| **AUROC**  (95%Cl)  p-value | 0.744  (0.678-0.810) p<0.0001 | 0.827  (0.769-0.884) <0.0001 | 0.877   (0.832-0.923)   <0.0001 | 0.898  (0.857-0.940)  <0.0001 | 0.911  (0.871-0.951)  <0.0001 |
| **Dichotomisation Probability cut-off** | high risk  >0.510 | high risk  >0.500 | high risk  >0.430 | high risk  >0.400 | high risk  >0.390 |
| **Sensitivity** (%) (95%Cl) | 55.9  (45-66) | 68.8  (58- 78) | 80.7  (71- 88) | 81.7  (72- 89) | 83.9  (75-91) |
| **Specificity** (%) (95%Cl) | 81.2  (73-88) | 80.3  (72 - 87) | 79.5  (71 - 86) | 80.3  (72 - 87) | 80.3  (72-87) |
| **PPV** (%)  (95%Cl) | 70.3  (61-78) | 73.5  (65- 80) | 75.8  (68 - 82) | 76.8  (69- 83) | 77.2  (70-83) |
| **NPV** (%)  (95%Cl) | 69.9  (64-75) | 76.4  (70 - 82) | 83.8  (77- 89) | 84.7  (78 - 90) | 86.2  (80-91) |

AUROC area under the roc curve, PPV, positive predictive value; NPV, negative predictive value.

**Table S4: Practical approach to predicting rapid progression to IA (n=210)**

| COX regression |  |  |  |  |
| --- | --- | --- | --- | --- |
|  | **Model ref** | **Model +**  **1 panel** | **Model +**  **2 panels** | **model +**  **3 panels** |
| Clinical  data | 2 clinical  Variables | 2 clinical variables | 2 clinical variables | 2 clinical variables |
| Flow panel -1  Treg |  | Treg CD4 | Treg CD4 | Treg CD4 |
| Flow panel -2  CD4/CD8 |  |  | Naïve CD4  CD4-IRC | Naïve CD4  CD4-IRC |
| Flow panel -3  lineage |  |  |  | CD8  NK CD56^dim^ |
| Accuracy  Nagelkerke's R^2^ | 0.179 | 0.322 | 0.400 | 0.45 |
| AUROC  (95%Cl)  p-value | 0.648  (0.644 0.652)  <.001 | 0.735  (0.732 0.738)  <.001 | 0.774  (0.771 0.777)  <.001 | 0.791  (0.788 0.793)  <.001 |

AUROC area under the roc curve. The 2 clinical variables included were RF and smoking.

Reference

15. Ponchel, F., Burska, A.N., Hunt, L., Gul, H., Rabin, T., Parmar, R., Buch, M.H., Conaghan, P.G. and Emery, P. T-cell subset abnormalities predict progression along the Inflammatory Arthritis disease continuum: implications for management. *Scientific Reports.* 2020, **10**(1), pp.3669-3669.

20. Ponchel, F., Burska, A.N., Hensor, E.M., Raja, R., Campbell, M., Emery, P. and Conaghan, P.G. Changes in peripheral blood immune cell composition in osteoarthritis. *Osteoarthritis Cartilage.* 2015, **23**(11), pp.1870-1878.
